# Immunotherapy Targeting Tumor-Associated Macrophages

**DOI:** 10.3389/fmed.2020.583708

**Published:** 2020-11-05

**Authors:** Yafei Liu, Rongsi Wang

**Affiliations:** ^1^Department of Pharmacy, The Forth Affiliation Hospital of China Medical University, Shenyang, China; ^2^High School of East China Normal University, Shanghai, China

**Keywords:** macrophage, tumor microenvironment, cancer, immunotherapy, polarization

## Abstract

Macrophages are phagocytic cells that play a broad role in maintaining body homeostasis and defense against foreign pathogens; whereas tumor-associated macrophages (TAMs) support tumor growth and metastasis by promoting cancer cell proliferation and invasion, immunosuppression, and angiogenesis, which is closely related to the poor prognosis in almost all solid tumors. Hence, deep-insight knowledge into TAMs can provide an opportunity to discover more effective strategies for cancer therapeutics. So far, a large number of therapeutic agents targeting TAMs are in clinical trials. In this review, we introduce an extensive overview about macrophages and macrophage-targeting agents.

## Introduction

Cancer, a global public health problem, is the first or second leading cause of death in most countries, and its incidence and mortality are rapidly growing (1). Clinically it is well-acknowledged that tumor sites contain not only cancer cells, but also immune cells, including macrophages, regulatory T (T_reg_) cells (2), neutrophils (3), mast cells (4), natural killer (NK) cells (5), etc. Macrophages, the main component of the mononuclear phagocyte system (6), are phagocytic cells which play a broad role in maintaining body homeostasis and defense against foreign pathogens; whereas there are a large number of TAMs in tumor microenvironment (TME), which support tumor growth and metastasis by promoting cancer cells proliferation, immunosuppression, invasion, and angiogenesis. Therefore, scientists pay special attention to TAMs when looking for effective cancer treatment strategies. In recent decades, several types of immunotherapies targeting TAMs are playing more and more important roles in the treatment of cancer.

This comprehensive review first summarizes most recent updates regarding macrophage recruitments and functions in tumor, then focuses on the development and evaluation of cancer immunotherapy strategies targeting TAMs including drugs in pre-clinical and clinical stages. Finally, we would like to provide some views and visions of immunotherapy targeting TAMs.

## Origins and Polarization of Macrophages

**Figure 1:**
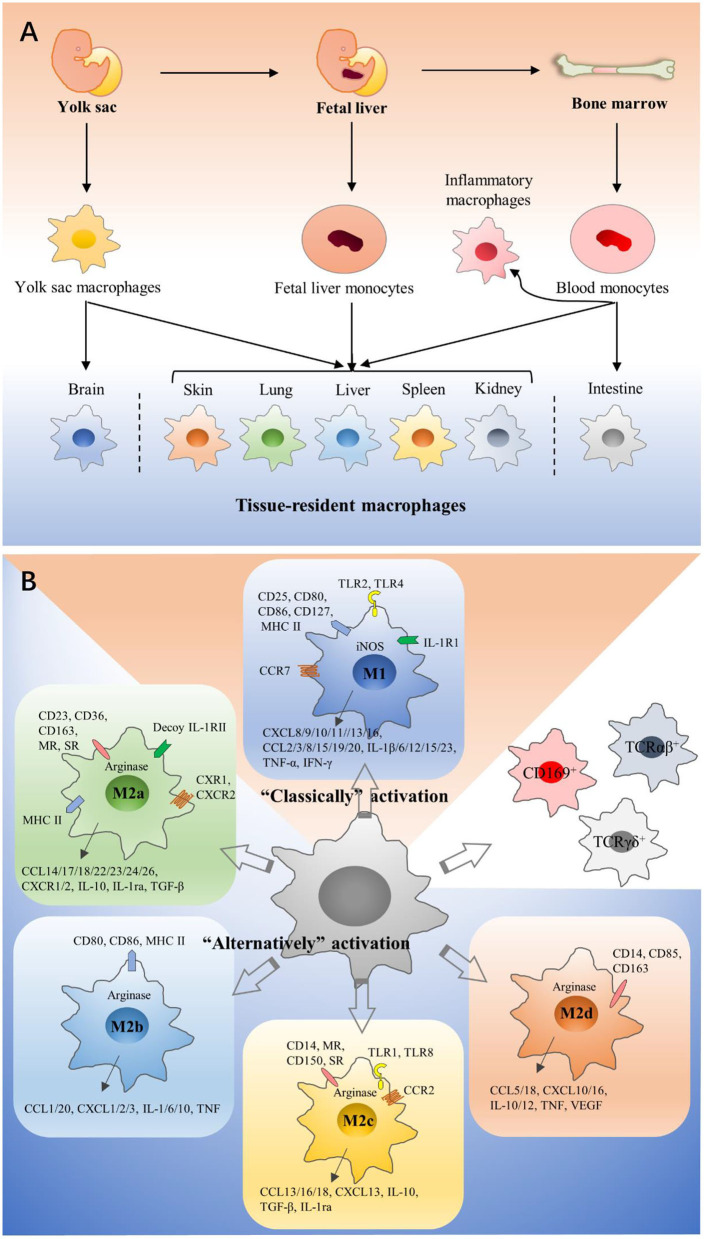
Origins and polarization of macrophages. **(A)** Macrophages can have three different developmental pathways: fetal yolk sac, fetal liver, and bone marrow. Precursors seed different tissues and differentiate into specialized tissue-resident macrophages on the basis of tissue-specific context, and they have dramatical differences in their phenotypes and functions. In tumors, TAMs are usually thought to primarily derive from circulating monocytes. **(B)** According to activation methods, macrophages are divided into M1 and M2 macrophages. M1 macrophages are polarized by LPS, which binds to TLR4. M2a macrophages are induced by IL-4 and IL-13. M2b macrophages are polarized by immune complexes and some TLR ligands. M2c macrophages would increase in the presence of IL-10 or glucocorticoids. M2d macrophages are induced by TLR agonists and adenosine. They have significant differences in surface receptor expression, metabolism, cytokine, and chemokine production. CD169^+^ macrophages, TCRαβ^+^, and TCRγδ^+^ macrophages are classified into neither M1 macrophages nor M2 macrophages.

Macrophages were first discovered and isolated by Ilya Metchnikoff in the nineteenth century (7). For decades, most people thought that blood-circulating monocytes derived from adult bone marrow (BM) continuously repopulate tissue-resident macrophages (TRMs). It is now well-accepted that a large number of TRMs derive from embryonic precursors, which are from both fetal yolk sac and fetal liver progenitors (8–12). All precursors seed different tissue and differentiate into specialized TRMs on the basis of tissue-specific context (10, 13). Moreover, most tissues also contain macrophages derived from monocytes after birth (13–15). However, some tissues are different, such that monocytes derived from hematopoietic stem cells (HSCs) fleetly take the place of embryonic macrophages after birth in the colon, but microglia are rarely from monocytes derived from HSCs under homeostatic conditions (16, 17) (Figure 1A). In tumors, TAMs are usually thought to primarily derive from circulating monocytes, and most recent studies have shown that functions and phenotypes of embryonic-derived and monocyte-derived macrophages are different (13, 18, 19). For example, Pierre-Louis Loyher et al. showed that embryonic-derived TAMs largely correlated with tumor cell growth *in vivo*, while monocyte-derived TAMs accumulation was associated with enhanced tumor spreading (18). Furthermore, several studies have suggested that TRMs are up to 50% in some murine models such as lung and brain cancer (18, 20).

Macrophages are a type of remarkable plastic cells and can be easily induced by surrounding microenvironment (21, 22). According to different activation methods, macrophages are divided into two extremes (23), Classically activated macrophages (M1 macrophages) and alternatively activated macrophages (M2 macrophages). M1 and M2 macrophages have significant differences in surface receptor expression, tissue distribution, metabolism, cytokine and chemokine production, function, and intracellular signal transduction. M1 macrophages are polarized by lipopolysaccharide (LPS), which binds to the Toll-like receptor 4 (TLR4). Then an inflammatory response is elicited (24), and pro-inflammatory cytokines are released, such as interleukin-1β (IL-1β), IL-6, and tumor necrosis factor-α (TNF-α). These downstream signals recruit more macrophages to resist pathogenic insult (25). M2 macrophages are polarized by cytokines such as IL-4 and IL-13, and release anti-inflammatory cytokines including transforming growth factor-β (TGF-β) and IL-10, inducing processes like membrane remodeling and angiogenesis to promote tissue repair (26, 27). Depending on specific inducing signals and their biological roles, M2 macrophages could be further divided into M2a, M2b, M2c, and M2d (28–32) (Figure 1B). Generally speaking, M1 macrophages mainly kill and clear cancer cells (33, 34), while M2 macrophages mainly support tumor development (35, 36). This M1/M2 concept can easily explain macrophage heterogeneity, but it is too simple to explain the complexity of macrophage activation. Actually, TAMs seem to consist of various populations with a wide range of polarization features or activation states, and their function is determined by microenvironment. Hence, additional studies are necessary to better classify macrophages, and there are several articles about other classifications (37–39).

## Functions of Macrophages in TME

### Promoting Tumorigenesis and Progression

TAMs are believed to be the bridge between cancer and inflammation. Some studies show that about 25% of all cancers are related to chronic infection and inflammation (40). The production of chemokines and cytokines are induced by key transcription factors [such as nuclear factor-κB (NF-κB)], hypoxia-inducible factor 1α (HIF1α), and signal transducer and activator of transcription 3 (STAT3) when chronic inflammation occurs, which activates the innate immune system and especially macrophages (41). There is a lot of evidence that the inflammatory microenvironment promotes genetic instability of tumor epithelial cells and tumor-infiltrating immune cells (42, 43). Recently, the inflammatory cytokines IL-23 and IL-17 secreted by TAMs have been shown to be closely related to human colorectal cancer progression (44). For instance, Kupffer cells can promote the progression of hepatocellular carcinoma by secreting mitogens, which relies on the NF-κB signaling pathway (45). Other results show that IL-6 produced by TAMs promotes the development of liver cancer through STAT3 signaling pathway (46), and IL-10 produced by TAMs promotes the development of non-small cell lung cancer through STAT1 signaling (47).

### Formation of the Immunosuppressive Microenvironment

Macrophages cannot only kill tumor cells directly when they are activated by interferon-γ (IFN-γ), but also recruit and activate CD8^+^ cytotoxic T lymphocytes and NK cells by presenting antigens and secreting cytokines to promote the adaptive immunity (48). In addition, T cells can activate monocytes through CD40-CD40L interplay to enhance their expression of major histocompatibility complex class II (MHC II), inducible nitric oxide (iNOS), and TNF (49). In fact, the T helper 2 (T_H_2) cells, dominating in the TME, activate macrophages to be polarized toward M2 macrophages, which promotes the development of immune suppression (50). Numerous studies have shown that TAMs can directly or indirectly inhibit T cell immune response through different mechanisms. The direct mechanisms include TAMs expressing inhibitory receptors to negatively regulate the activation of T cells by interaction with CD94 (51), expressing T cell immune checkpoint ligands to inhibit T cell functions (52, 53), producing cytokines to maintain a immunosuppressive microenvironment through inducing T_reg_ cell expansion and inhibiting CD4^+^ and CD8^+^ T cells (54, 55), and depleting L-arginine and tryptophan to inhibit cytotoxic T cells (56, 57). The indirect mechanisms include TAMs regulating the release of chemokines to control the recruitment of T_reg_ cells (58, 59), and blunting T cell recruitment by regulating the extracellular matrix (ECM) (60).

### Promoting Invasion and Metastasis

Cancer metastasis is a complicated event, which plays a crucial role in the cause of morbidity and mortality (61, 62). It is worth noting that macrophages play an important role in tumor cells invasion and metastasis. They facilitate the escape of tumor cells from the basement membrane through the dense stroma by producing proteases to promote ECM degradation (63). Furthermore, several factors, such as macrophage-colony stimulating factor (M-CSF/CSF-1), can stimulate macrophages to promote tumor invasion (64, 65). Metastasis-associated macrophages (MAMs), a unique population of macrophages, have been identified are found to be recruited by CC chemokine ligand (CCL) 2 (66, 67). MAMs promote cancer cell invasion and metastasis by FMS-like tyrosine kinase 1 (FLT1) receptor tyrosine kinase signaling in a mouse model of breast cancer (68). In addition, several studies show that the activation of the CCL2/CC chemokine receptor (CCR) 2 axis is very important in MAM-mediated metastasis (66, 67, 69). Recent studies have shown that pre-metastatic niche (PMN) is a pre-requisite in mediating tumor cell metastasis. Primary tumor cells are thought to initiate the formation of PMN by the secretion of proinflammatory cytokines, chemokines, and angiogenic factors that recruit BM-derived cells into future metastatic sites, and these cells induce PMN formation in reverse (70). For example, CXCL1 secreted by TAMs was reported to recruit CXCR2^+^ myeloid suppressor cells to promote liver PMN formation (71, 72).

### Promoting Angiogenesis

Angiogenesis is necessary for tumor growth and metastasis, which is regarded as a “hallmark” of cancer (73). Accumulating evidence emphasizes the crucial roles of macrophages in promoting tumor angiogenesis, and TAMs is closely related to the number of blood vessels in the tumor (74). Hypoxia is the primary driver of angiogenesis, and some studies show that anoxic areas of tumors, especially the necrotic tissue, have large numbers of macrophages due to the releasing of endothelins, vascular endothelial growth factor (VEGF), high mobility group 1, CCL2, CXC chemokine ligand 8 (CXCL8), CXCL12, and CSF-1 (75). The increased expression of hypoxia-inducible transcription factors on TAMs up-regulates the transcription of various genes in hypoxic tumor sites, which responds to hypoxia and promotes tumor cells proliferation, metabolism, and angiogenesis (75–77). In a CSF-1 knockout mice model, macrophage number was found to significantly reduce in the tumor site, accompanied by impaired vascular development (78). In addition, tumor endothelium-released angiopoietin-2 (TIE-2) was reported to play an significant role in tumor angiogenesis by recruiting monocytes that express the TIE-2 receptor (79). Furthermore, results of gene analysis indicated that TAMs could up-regulate the expression of various factors, which participate in tumor angiogenesis and provide nutrients for tumor growth (39).

## Immunotherapy-Targeting TAMS in Cancer

### Restoration of Macrophage Phagocytosis

**Figure 2:**
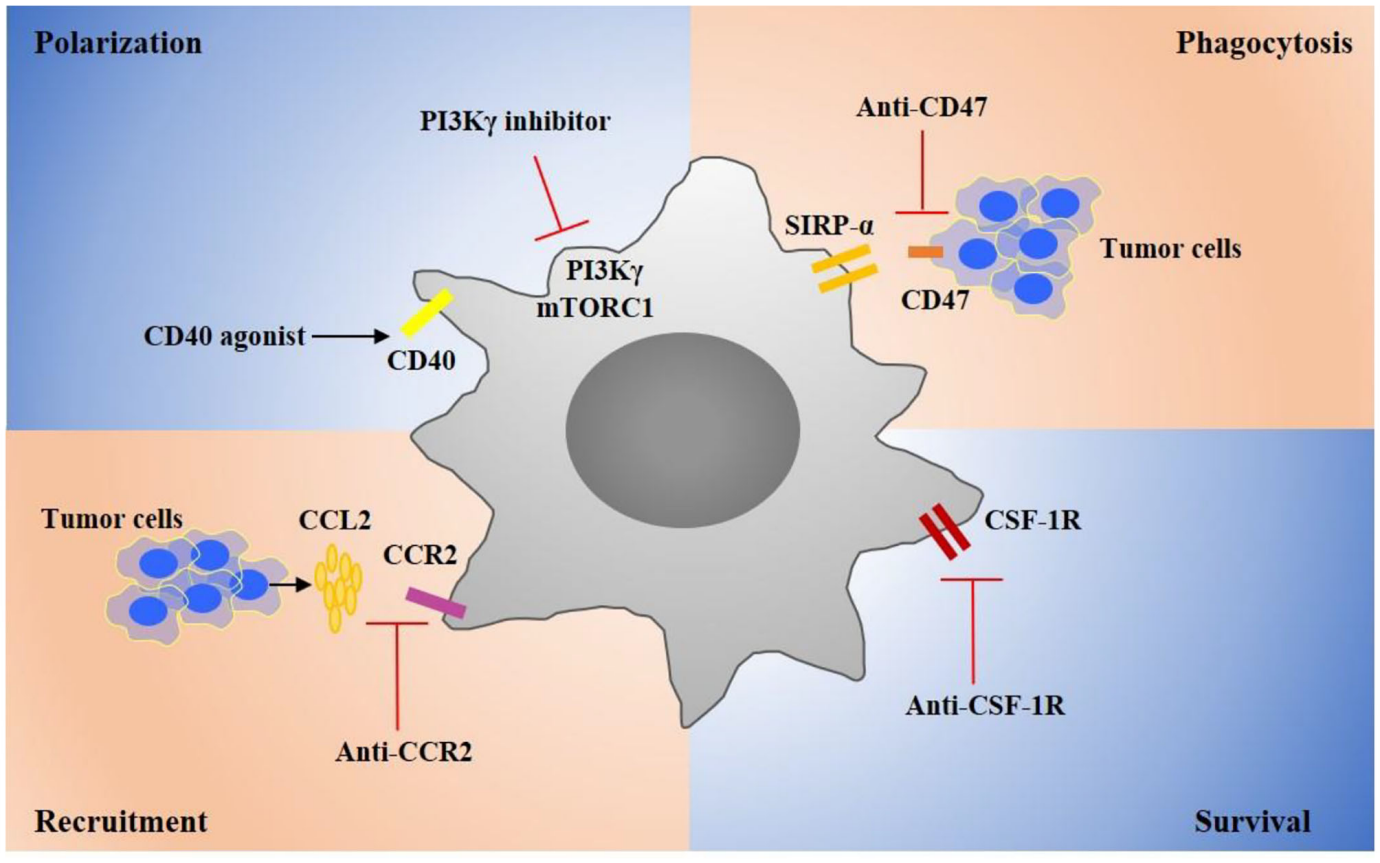
Targeting TAM strategies in cancer treatments. Several critical targets have been identified that regulate TAMs recruitment, polarization, survival, and phagocytosis during tumor progression. Targeting key receptors or signaling molecules can modulate these macrophage properties and suppress tumor progression. For example, targeting CSF1R can suppress the survival of TAMs. Agonists of CD40 can promote TAMs toward a proinflammatory phenotype that can suppress tumor. Inhibitors of CCR2 or CXCL2 can inhibit monocyte recruitment. Targeting CD47 on tumor cells can promote macrophage phagocytosis of tumor cells. These therapeutic strategies are developed to promote effective antitumor immune responses and many drug candidates are currently investigated in clinical trials for cancer therapy.

CD47 has been found expressed on many tumor cells, and it can bind with signal regulatory protein α (SIRPα) on the membrane surface of macrophages, which down-regulates macrophage phagocytosis of tumor cells (80, 81). In the past few years, a number of clinical trials have been conducted to determine various treatments that block CD47/SIRPα (Figure 2) (82). Anti-CD47 antibody treatment could inhibit tumor growth in a pediatric brain malignancies model (83). Anti-CD47 antibody in combination with TTI-621, a SIRPα-Fc fusion protein that could block the binding between SIRPα and CD47, promotes phagocytosis of tumor cells in s B-cell lymphoma mouse model (84). Hu5F9-G4, a human monoclonal antibody directing against CD47 has been tested in a tumor therapy as a single agent, as well as in combination with cetuximab. Nevertheless, anti-CD47 therapies may increase the occurrence of transient anemia, because HSCs and red blood cells extensively express CD47 (85, 86). Furthermore, there are other “don't eat me” signals including programmed cell death ligand 1 (PD-L1), MHC 1 component β2-microglobulin, and CD24, and antibodies which direct against the interaction of these signals with their macrophage surface receptors have demonstrated therapeutic potential in several cancers (87–89).

### Inhibition of Macrophage Recruitment

Under tumor microenvironment, monocytes are rapidly recruited into tumor (90). Chemokines CCL2, CCL3, CCL4 and cytokines IL-1β, and CSF-1 have proven to contribute to the monocyte recruitment into tumors (91, 92). It is shown that CCL2 expression is up-regulated by macrophages and tumor cells in TME (93–95). Moreover, the high expression of CCL2 has a correlation with the poor prognosis in many human and murine tumors (96). CCL2 promotes monocytes recruitment by stimulating CCR2. In fact, blocking CCL2/CCR2 not only inhibits the monocyte infiltration but also prevents immunosuppressive polarization of macrophages (97, 98). Currently, a number of treatments targeting CCL2/CCR2 are in clinical trials (99) (Figure 2). A CCR2 inhibitor, PF-04136309, has been demonstrated to effectively inhibit tumor growth in pancreatic cancer patients (100, 101). CCL2 antibody treatment has proven to suppress tumor metastasis in a breast cancer model (96). Moreover, IL-1β has been identified as a chemoattractant target for cancer treatment. An IL-1 receptor antibody has been demonstrated to suppress inflammatory macrophage accumulation and tumor growth in lung and breast cancer mouse models (100). Moreover, in combination with fluorouracil and bevacizumab, Anakinra, an IL-1 receptor antibody, has been shown to prolong patients' life in a colorectal carcinoma Phase II clinical trial (102) (Table 1).

**Table 1:** Clinical trials of macrophage-targeting agents.

	**Drug**	**Company**	**Clinical trial number**	**Tumor type**	**Phase**
CD47	Hu5F9-G4	Forty Seven	NCT02953782	Advanced solid malignancies and colorectal carcinoma + cetuximab	I
			NCT02216409	Advanced solid malignancies	I
	TTI-621	Trillium	NCT02663518	Small cell lung cancer	I
			NCT02890368	Relapsed and refractory solid tumors	I
CD40	SEA-CD40	Seattle Genetics	NCT02376699	Solid tumors + pembrolizumab	I
	APX005M (Agonist antiCD40)	Apexigen	NCT03389802	Pediatric CNS	I
	CP-870,893 (agonist antiCD40)	VLST Corporation	NCT01103635	Metastatic melanoma + tremelimumab (antiCTLA-4)	I
	R07009879 (selicrelumab, agonist antiCD40	Roche	NCT02760797	Advanced solid tumors + anti-PDL1	I
			NCT02665416	Advanced solid tumors + bevacizumab or vanucizumab	I
			NCT02588443	PDAC + gemcitabine + nab-paclitaxel	II
CSF1R	BLZ945	Novartis	NCT02829723	Advanced solid tumors single agent	I
				Advanced solid tumors + PDR001	II
	Emactuzumab	Hoffman La Roche	NCT02323191	Advanced solid tumors + atezolizumab	I
			NCT03708224	Advanced HNSCC + atezolizumab	I
			NCT03193190	PDAC + additional therapies	I
	IMC-CS4 (antiCSF1R)	Lilly	NCT01346358	Advanced solid tumors	I
			NCT02265536	Advanced breast, prostate cancer	I
			NCT03153410	PDAC + cyclophosphamide pembrolizumab, GVAX	I
CCR2	BMS-813160	Bristol Meyers Squibb	NCT02471716	Tenosynovial giant cell tumor	II
			NCT03158272	Advanced malignancy + nivolumab	I
			NCT02526017	Advanced solid tumors + nivolumab	I
	CCX872-B	ChemoCentryx	NCT03778879	PDAC + SBRT	II
	MLN1202 (antiCCR2 antibody)	Millennium	NCT01015560	Bone metastases	II
IL1Ra	Anakinra	Swedish Orphan Biovitrum	NCT0255032	7 PDAC + abraxane, gemcitabine, cisplatin	I
TLR4	GSK1795091	GlaxoSmithKline	NCT03447314	Advanced solid tumors + GSK3174998 antiOX40) or (GSK3359609 anti-ICOS) or pembrolizumab	I
Stat3	TTI-101	Tvardi Therapeutics	NCT03195699	Advanced cancers	I
PI3Kγ	IPI-549	Infinity Pharmaceuticals	NCT02637531	Advanced solid tumors + nivolumab	Ib
BTK	Ibrutinib	Pharmacyclics/AbbVie	NCT02599324	Renal cell, urothelial, gastric, colon, pancreatic adenocarcinoma	III
			NCT02436668	PDAC, gemcitabine + nab-paclitaxel	Ib/II
			NCT02403271	Relapsed or refractory solid tumors + durvalumab	III

### Controlling Macrophage Proliferation and Survival

CSF-1 receptor (CSF-1R), a tyrosine kinase receptor, plays a key role in regulating macrophage proliferation and survival (103). Several studies show that blocking CSF-1/CSF-1R inhibited immunosuppressive macrophage polarization, reduced tumor cell proliferation, and promoted apoptosis, therefore suppressing tumor progression and prolonged life survival (104, 105) (Figure 2). M279, a CSF-1R antibody, blocking both CSF-1 and IL-34, has been shown to inhibit tumor growth and improve survival rate in a spontaneous breast tumor model (106, 107). BLZ945, a small-molecule CSF-1R inhibitor has been reported to be therapeutically effective in glioma and breast cancer mouse models (108). Moreover, a number of CSF-1R-specific inhibitors, including PLX3397, PLX7486, and BLZ945, have been tested in clinical trials (109, 110). Especially, PLX3397, exhibiting higher affinity to CSF-1R, has demonstrated a better effect for tenosynovial giant cell tumor therapy, and the drug has been advanced into clinical trial phase III (111). In addition, several FDA-approved tyrosine kinase inhibitors, such as targeting c-KIT and VEGFR, have also been shown to have a binding activity with the CSF-1R kinase (112).

### Modulation of Macrophage Phenotype

PI-3 kinase γ (PI3Kγ) has been identified as a promising target for modulating macrophage phenotype and proinflammatory cytokine expression (113) (Figure 2). IPI-549, a PI3Kγ inhibitor, is currently tested in Phase 1b clinical trials for several solid tumors, in combination with nivolumab. Bruton's tyrosine kinase (BTK), a downstream of PI3Kγ, has been investigated as a target for cancer treatment. In line with studies, ibrutinib, a BTK inhibitor, has been advanced in Phase III clinical trials for pancreatic adenocarcinoma treatment and in Phase II clinical trials for relapsed or refractory solid tumor therapy in combination with durvalumab. Janus kinase 2 (JAK2) and STAT3 also have been regarded as potential targets for macrophage repolarization (114). The STAT3 inhibitor TTI-101 is currently investigated in a Phase I clinical trial for advanced cancers, and the JAK2 inhibitor has been applied for the treatment of psoriasis, myelofibrosis, and rheumatoid arthritis in clinic (115).

CD40 is mainly expressed on antigen presenting cells, monocytes, and some tumor cells. CD40 ligation in macrophages induces secretion of proinflammatory cytokines and promotes macrophage polarization toward a proinflammatory macrophage. Several anti-CD40 antibodies and CD40 ligands, such as RO7009789, APX005M, are currently under test and evaluation in clinical trials for solid tumors (Figure 2). Interestingly, unlikely other activatory Fc receptors, the antibody Fc domain with inhibitory FcγRIIb is required for anti-40 antibody because of its agonistic immunostimulatory activity. In particular, CP-870893, a Pfizer anti-CD40 antibody of IgG2 subclass, has been shown to be more competitive in immunostimulation compared to other drugs in clinical trials (116). Moreover, TLR agonist treatment has been studied and developed for cancer therapy because TLRs stimulation can polarize macrophages toward a proinflammatory phenotype.

### Metabolic Modulation of TAMs

To support specialized cellular activities, macrophages use diverse metabolic pathways for energy and metabolite at different states (117). Metabolic changes contribute to the regulation of macrophage polarization, and TAMs display an immunosuppressive phenotype that is defined by the production of ornithine and polyamines through the arginase pathway as well as by expression of T_H_2 cytokines that include IL-10 (118–120). Several studies have shown that the tumor microenvironment, featured poor nutrient and acidic environment, directly induced macrophages to adopt immunosuppressive phenotypes (121–123). For example, lactate, a byproduct of tumor cells, can promote monocytes and macrophages toward to immunosuppressive macrophage polarization in B16 melanoma and lung carcinomas mouse model (121). Moreover, the tumor microenvironment in melanomas characterized by acid has been reported to promote immunosuppressive polarization of TAMs, including upregulating arginase and VEGF expression (124). Collectively, these studies have shown that altering the metabolic pathways of TAMs to repolarize macrophages might be an effective strategy for antitumor functions.

The PI3K/Akt/mTOR myeloid signaling pathway plays a key role in regulation of TAMs metabolism by promoting L-arginine metabolism, a curial section that could promote immunosuppression. The gene and protein expression of Arginase-1 (Arg-1) in TAMs up-regulates and inhibition of PI3Kγ can suppress Arg-1 expression and activity (90). Additionally, the deletion of PI3Kγ promotes the expression of the enzyme NOS, which promotes the production of the free radical and NO to function as anti-tumor. Kaneda et al. reported that IPI-549, a PI3kγ inhibitor, inhibited lung carcinoma and breast tumors by promoting TAM-immunostimulatory response (125). Moreover, mTORC1 and mTORC2 also play a key role in the metabolic programming of macrophages by sensing nutrients, oxygen, and metabolites. Rapamycin, an mTORC1 inhibitor, has been reported to promote macrophages toward the proinflammatory phenotype with an anti-tumor effect (126) (Figure 2).

### Adoptive Macrophages Transfer

Adoptive cell transfer is an emerging method of immunotherapy, which kills and removes cancer cells by the infusion of immune cells (127). Macrophages have the capacity to penetrate tumors (128), which may kill tumor cells where CAR-T therapy has fallen (129). Therefore, adoptive macrophage transfer (AMT) has become a hot research field for tumor detection and treatment lately. Amin Aalipour et al. used engineered macrophages as diagnostic sensors to successfully detect tumors as small as 4 mm in diameter and show better sensitivity than traditional cancer biomarkers (130). Recently, Michael Klichinsky et al. described an anti-HER2 CAR-macrophage (CAR-M) that significantly reduced metastatic tumor burden (131). A cellular IFN-γ “backpack” for macrophages was reported to promote phagocytosis and polarize macrophages toward the M1 phenotype, which further slows down the tumor growth in a murine breast cancer model (132). Overall, the adoptive transfer therapy of macrophages is still in the research stage, and there are many problems to be solved, such as the establishment of pre-clinical models to evaluate the efficacy and safety of AMT. In addition, the way to efficiently transfer genes into human macrophages is still challenging and needs further study.

## Discussion

Various strategies targeting TAMs have been studied for cancer therapy, and some treatments have been advanced into clinical trials. However, because of complexity of tumors, a combination therapy is usually adopted to maximize the anti-tumor effect; whether currently targeted signaling pathways therapeutically overlap or synergize *in vivo* remains to be explored. More importantly, current researches do not have a thorough understanding of these targets, and their other functions are often overlooked in cancer treatment. Besides, with multiple targets being identified and drugs being tested for the modulation of TAMs, drug delivery technologies have been advanced to further enhance the efficacy of these drugs, through the way of improving stability, selectivity, and intracellular delivery efficiency, etc. CAR-M, as an emerging strategy for cancer therapeutic, is still in research stage. Currently, overcoming the challenge that genes transfer into human macrophages and finding effective solid tumor targets are the main tasks. Perhaps CAR-M in the future is to adopt multiple macrophages having different functions rather than a single population.

TAMs represent a heterogeneous population with different functions according to different origins and contexts. Consequently, it is necessary to understand this heterogeneity and how it evolves during the progression of cancer and also following therapy in human, not mouse, models. In this context, the extensive use of single-cell RNA sequencing, multiplex immunohistochemistry, and mass cytometry will considerably increase our knowledge about TAMs, which is essential for the adoption of precision medicine and good prediction of patient responses. Admittedly, many questions remain regarding to properties and functions of macrophages in TME. However, with the deeper understanding of macrophage diversity through single-cell sequencing and other technologies, we believe that TAM-targeted treatment will be an important addition for cancer immunotherapy.

## Author Contributions

YL conceived the concept and wrote the manuscript. RW edited and improved the manuscript. All authors contributed to the article and approved the submitted version.

## Conflict of Interest

The authors declare that the research was conducted in the absence of any commercial or financial relationships that could be construed as a potential conflict of interest.

## Abbreviations

AMT: adoptive macrophages transfer
Arg-1: arginase-1
BM: bone marrow
BTK: Bruton's tyrosine kinase
CAR-M: chimeric antigen receptor macrophage
CAR-T: chimeric antigen receptor T cells
CCL: CC chemokine ligand
CCR: CC chemokine receptor
CSF-1R: CSF-1 receptor
CXCL8: CXC chemokine ligand 8
ECM: extracellular matrix
FLT1: FMS-like tyrosine kinase 1
HIF1α: hypoxia-inducible factor 1α
HSCs: hematopoietic stem cells
IFN-γ: interferon-γ
iNOS: inducible nitric oxide
Jak2: Janus kinase 2
IL-1β: interleukin-1β
LPS: lipopolysaccharide
MAMs: metastasis-associated macrophages
M-CSF/CSF-1: macrophage-colony stimulating factor
MHC II: major histocompatibility complex class II
MPS: mononuclear phagocyte system
MR: mannose receptor
NF-κB: nuclear factor-κB
NK: natural killer
PI3Kγ: PI-3 kinase γ
PMN: pre-metastatic niche
SIRPα: signal regulatory protein α
SR: scavenger receptor
STAT3: signal transducer and activator of transcription 3
TAMs: tumor-associated macrophages
TGF-β: transforming growth factor-β
T_H_2: T helper 2
TIE-2: tumor endothelium releases angiopoietin-2
TLR4: Toll-like receptor 4
T_reg_: regulatory T
TRMs: tissue-resident macrophages
TME: tumor microenvironment
TNF-α: tumor necrosis factor-α
VEGF: vascular endothelial growth factor.
